# Stress Wave Signal Denoising Using Ensemble Empirical Mode Decomposition and an Instantaneous Half Period Model

**DOI:** 10.3390/s110807554

**Published:** 2011-08-02

**Authors:** Yi-Ming Fang, Hai-Lin Feng, Jian Li, Guang-Hui Li

**Affiliations:** School of Information Engineering, Zhejiang A & F University, Hangzhou, Zhejiang, 311300, China; E-Mails: ilone_fang@126.com (Y.-M.F.); lsword402@yahoo.com.cn (J.L.); lgh@zafu.edu.cn (G.-H.L.)

**Keywords:** ensemble empirical mode decomposition, denoising, instantaneous half period, stress wave, wood test

## Abstract

Stress-wave-based techniques have been proven to be an accurate nondestructive test means for determining the quality of wood based materials and they been widely used for this purpose. However, the results are usually inconsistent, partially due to the significant difficulties in processing the nonlinear, non-stationary stress wave signals which are often corrupted by noise. In this paper, an ensemble empirical mode decomposition (EEMD) based approach with the aim of signal denoising was proposed and applied to stress wave signals. The method defined the time interval between two adjacent zero-crossings within the intrinsic mode function (IMF) as the instantaneous half period (IHP) and used it as a criterion to detect and classify the noise oscillations. The waveform between the two adjacent zero-crossings was retained when the IHP was larger than the predefined threshold, whereas the waveforms with smaller IHP were set to zero. Finally the estimated signal was obtained by reconstructing the processed IMFs. The details of threshold choosing rules were also discussed in the paper. Additive Gaussian white noise was embedded into real stress wave signals to test the proposed method. Butterworth low pass filter, EEMD-based low pass filter and EEMD-based thresholding filter were used to compare filtering performance. Mean square error between clean and filtered stress waves was used as filtering performance indexes. The results demonstrated the excellent efficiency of the proposed method.

## 1. Introduction

Stress-wave-based techniques have been investigated extensively and have shown promise for predicting the mechanical properties of wood materials, such as small, clear wood specimens, lumber, veneers, and wood based composites [1]. During the past few decades, computerization of the stress wave process has been sought, supported by advances in computers and technology along with advanced signal processing methods. Many researchers have developed algorithms improving the accuracy and expanding the application field. Wavelet and spectral analysis were used to distinguish various types of distorting transient vibrations and associated stress wave propagation modes. Cubic spline wavelet analysis was utilized to localize the dominant stress wave velocities [2]. Bozhang and Pellerin transformed the stress wave signal from the time domain to the frequency domain by Fast Fourier transformation; then the incipient decay can be identified by measuring the stress wave signal frequency [3]. Recent research has focused on stress wave tomography to provide clear interior features of wood and wood defect images [4,5]. Besides these, there have been efforts to understand the propagation behavior by computer simulation methods. In our previous work [6,7], the propagation of stress waves in anisotropic elastic media like wood was studied according to mechanical wave theory in solids.

Even so, until now, no developed method or system is used worldwide. It is difficult to extract useful information directly from the raw stress wave signals, which are nonlinear, non-stationary and are often corrupted by noise. In practice, when the hammer hits the wood, the generated stress waves are always followed by a mixture of dilatational waves and shear distortions. The collected signals can be viewed as the result of multiple interferences and reflections of these two waves fitting the boundary conditions, which interfered with the stress wave information identification [8]. Therefore, noise reduction is a necessary step for any stress wave based wood test technique to paves the way for further discovery in physics and nature.

Unfortunately, classical noise reduction methods are based on spectrum analysis and trend to induce a relative big phase delay which will result in an inaccurate evaluation of the tree’s internal condition. Wavelet based denoising method removes noise from received signals by threshold operations on wavelet coefficients and its efficiency has been confirmed [9], however, it’s difficult to select the wavelet base, scale, threshold function and optimal threshold value. Therefore, it is also not desirable for stress wave denoising.

The empirical mode decomposition (EMD) algorithm is a technique designed by Wu and Huang primarily for decomposing the nonlinear and non-stationary signals into a series of intrinsic mode functions (IMFs) [10]. It has been used to address several problems in the field of science and engineering [11]. The property of EMD to behave as a dyadic filter bank resembling those involved in wavelets [12] has been useful in signal denoising. Some of the typical applications are represented in the literature [11,13–17]. In 2009, ensemble EMD (EEMD) was introduced to remove the mode-mixing effect [18]. As a more robust and noise-assisted version of EMD, it was also used in noise reduction [19–21]. By adding finite white noise to the investigated signal, the EEMD method can eliminate the problem of mode mixing automatically to improve EMD [22]. Consequently, EEMD can achieve better filtering performance than EMD with suitable added noise and a sufficient number of trials. Although EEMD has a heavy computational load, it is still suitable for getting better noise reduction performance [19,20].

The existing noise reduction methods using EMD or EEMD can be divided into two categories. In this work, we call them EEMD-based thresholding filter [14,16] and EEMD-based low pass filter [13,17,19]. EEMD-based thresholding filter reconstructs the signal with all the IMFs that were previously thresholded as in wavelet analysis. Due to the fact that most of the important structures of the signal are often concentrated in lower frequency components (high order IMFs) and decrease toward the high frequency modes (low order IMFs), the noise power can be suppressed significantly by applying a suitable threshold on the high frequency modes. However, when applying the threshold on the high order IMFs, which contain little or no noise, the main signal features may be changed. The second approach, EEMD-based low pass filter, was developed based on the assumption that the IMFs derived by EEMD will only be divided into two classes: noise-only IMFs and signal-only IMFs. Then we can use a criterion to classify and remove the noise-only IMFs. Only the signal-only IMFs are partially reconstructed. However, noises are usually distributed over all IMFs. Thus the low pass scheme of the signal removes the high-frequency components of both the noise and the signal and leaves the low-frequency components of noise.

In this paper, an interesting stress wave filtering method based on EEMD was proposed aiming to obtain an improved stress wave signal with reduced artifacts. This work was done as a preprocessing step for computerized nondestructive wood testing using the stress wave technique.

## 2. EMD and EEMD Algorithm

### 2.1. EMD Algorithm

The EMD algorithm can be described as follows [10]:

1. Extract all the local maxima and minima of *x*(*k*) .
2. Form the upper and lower envelop by cubic spline interpolation of the extrema point developed in step (1).
3. Calculate the mean function of the upper and lower envelop, *m*_1_(*k*).
4. Let *h*_1_(*k*) *= x*(*k*) *− m*_1_(*k*). If *h*_1_(*k*) is a zero-mean process, then the iteration stop and *h*_1_(*k*) is an IMF1, named it as *c*_1_(*k*), else go to step (1).
5. Define *r*(*k*) *= x*(*k*) *− c*_1_(*k*).
6. If *r*(*k*) still has least 2 extrema then go to step (1) else decomposition process is finished.

At the end of the procedure, we have a residue *r*(*k*) and a collection of *n* IMFs, named from *c*_1_(*k*) to *c_n_*(*k*). The original signal can be represented as:

(1) $$x(k)=\sum_{i=1}^{n}c_{i}(k)+r(k)$$

Note that the EMD dose not use any predetermined basis functions or does not require any user parameter setting. It is a fully data-driven method. Consequently, the results preserve the full nonstationarity characteristics of the stress wave signals. Seen in this light, the EMD method is superior to the wavelet analysis approach, where the basis functions are fixed and, thus, do not necessarily match all real signals [13]. In addition, the selection of the wavelet is too vital to make the method strict [23].

### 2.2. EEMD Algorithm

One of the major drawbacks of the EMD algorithm is the appearance of mode mixing. Therefore the EEMD algorithm was introduced. The algorithm defines the IMF set for an ensemble of trials, each one obtained by applying EMD to the signal of interest with added independent identically distributed white noise of the same standard deviation. Taking into account properties of the white noise, the problem of mode mixing can be overcome.

The steps for EEMD are as follows [18]:

1. Initialize the number of ensemble *M*, the amplitude of the added white noise and *m =* 1.
2. Add a white noise series to the targeted signal, *x_m_*(*k*) *= x*(*k*) *+ n_m_*(*k*).
3. Apply EMD to the noise-added signal *x_m_*(*k*) to derive a set of IMFs *c_i,m_*(*k*) (*i =* 1, 2,*…*,*n*), where *c_i,m_*(*k*) denotes the *i^th^* IMF of the *m^th^* trial and *n* is the number of IMFs.
4. Repeat steps (1) and (2) until *m > M*.
5. Average over the ensemble to obtain the final IMF of decompositions as the desired output:
  (2) $$\bar{c_{i}}(k)=\frac{1}{M}\sum_{m=1}^{M}c_{i,m}(k),i=1,2,\cdots n$$

## 3. The Proposed Denosing Approach

### 3.1. Definition of Instantaneous Half Period (IHP)

Let *c_i_*(*k*) denote the *i^th^* IMF, *i =* 1, 2,*…*,*n*, where *n* is the number of IMFs. Then the zero-crossings of *c_i_*(*k*) can be located by mathematical operations. We named this as ${\mathrm{ZP}}_{i}^{j}$, which denotes the *j^th^* zero-crossing of the *i^th^* IMF. Accordingly, the time when ${\mathrm{ZP}}_{i}^{j}$ emerges is defined as $\tau_{i}^{j}$. Hence we can treat the time interval between ${\mathrm{ZP}}_{i}^{j+1}$ and ${\mathrm{ZP}}_{i}^{j}$ as the half period of an oscillation. Considering the half periods may be different with each other, we define it as IHP and compute it as follows:

(3) $$T_{i}^{j}=\tau_{i}^{j+1}-\tau_{i}^{j}$$

**Figure 1.**
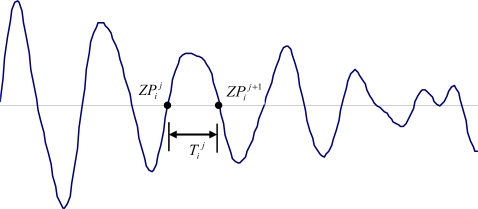
Illustration of zero-crossings and IHP model defined in this study.

Examples of zero-crossings and IHP were plotted in the Figure 1. The waveform shown in the Figure 1 is a part of the *i^th^* IMF. If one of the zero-crossings is numbered as ${\mathrm{ZP}}_{i}^{j}$, then the next zero-crossing is ${\mathrm{ZP}}_{i}^{j+1}$, and the time interval, $T_{i}^{j}$, between the ${\mathrm{ZP}}_{i}^{j+1}$ and ${\mathrm{ZP}}_{i}^{j}$ is the *j^th^* IHP of the *i^th^* IMF.

### 3.2. Threshold Operation According to the IHP

Generally, the signal structures correspond to the slow time variation of data and the frequency is often lower than the noise structures [13]. Thus, one can assume that, the IHP of a signal dominated oscillation is longer than the IHP of a noise dominated oscillation. According to this idea, there will be a threshold *thr*, which allows us to retrieve the most important structures of the signal from its noisy version. The waveforms between the two adjacent zero-crossings will be considered as signal dominated oscillations and be retained. Whereas the waveforms with smaller LHP will be treated as noise dominated oscillations and be set to zeros. This process can be described as:

(4) $${\hat{c}}_{i}(k)=\{\begin{array}{c} c_{i}(k),\;\;\;T_{i}^{j}\geq \mathrm{thr}\\ 0,\;\;\;\;\;\;\;\;\mathrm{others} \end{array},{\mathrm{ZP}}_{i}^{j}<k\leq {\mathrm{ZP}}_{i}^{j+1}$$

Finally a reconstruction process of projecting the restored IMF, *ĉ_i_*(*k*), back onto the filtered signals is done as follows:

(5) $$\hat{x}(k)=\sum_{i=1}^{n}{\hat{c}}_{i}(k)+\hat{r}(k)$$

### 3.3. Selection of the Optimum Threshold

Selection of the optimum threshold value *thr* also plays an important role in the proposed method. A large *thr* would result in oversmoothing of the target signal, thus removing some low-frequency oscillations while these oscillations are signal dominated. Moreover a small *thr* might not be able to remove the artifacts, hence resulting in a signal of relatively low quality.

Here we suggest two methods to select the optimum threshold value. On condition that the frequency range of the target signal is known, the optimum threshold value can be obtained by the maximum frequency:

(6) $${\mathrm{thr}}_{\mathrm{opt}}=\frac{\alpha }{2f_{h}}$$

where *f_h_* denotes the maximum frequency of the target signal, *α* is a constant coefficient used to determine the frequency range of the retained oscillation. When *α* = 1, the maximum frequency of retained oscillation equates the maximum frequency of the target signal.

If the *a priori* knowledge of the target signal is unknown, the optimum threshold value can be determined by experiments. A cost function, *J*(*thr*), can be defined by utilizing a measurement of denoising performance, such as mean square difference (MSD) [24]. A small value implies a better performance. Consequently the optimal threshold value is given by:

(7) $${\mathrm{thr}}_{\mathrm{opt}}=\text{arg}\;\;\text{min}\{J(\mathrm{thr})\}$$

In this work the first method is used. Due to the fact that the frequency of the stress wave signal ranges from 0 to 5 KHz, the optimum threshold value can be set by *thr_opt_* *=* 0.1*α* ms.

### 3.4. The Operation Procedures of the Proposed Method

**Figure 2.**
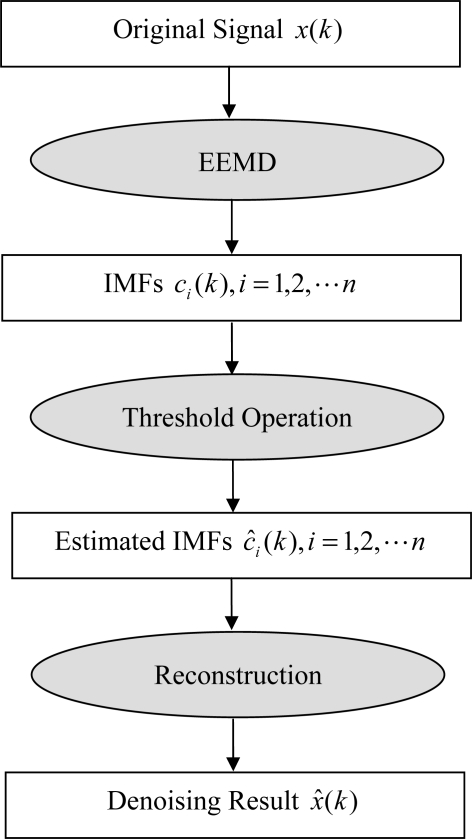
The operation procedures of the proposed denoising method.

The operation procedures of the proposed denoising method can be summarized in the flowchart as shown in Figure 2.

1. Apply EEMD to the original signal *x*(*k*) to extract a set of IMFs *c_i_*(*k*) (*i =* 1, 2,*…*,*n*).
2. Compute *thr_opt_*, using Equation (6).
3. Apply the threshold operation to each IMF using Equation (4).
4. Reconstruct *x̂*(*k*), which is the filtered signal, using Equation (5).

## 4. Results and Discussions

### 4.1. Stress Wave Signals and Noises Preparation

**Figure 3.**
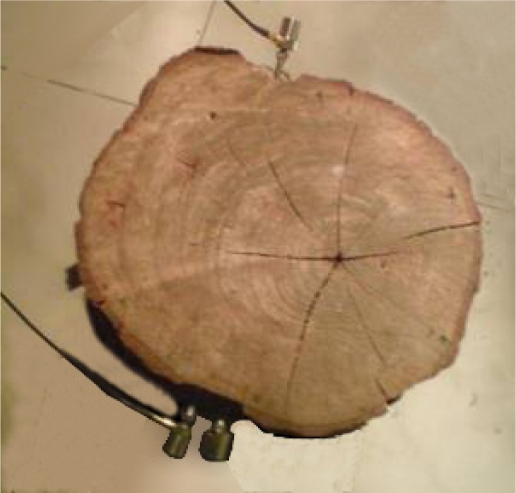
The *Cinnamomum camphora* sample used in the experiment.

**Figure 4.**
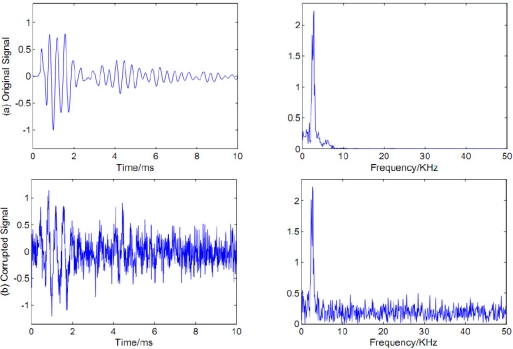
A typical stress wave signal and its corrupted version. From top to bottom: **(a)** Original signal. **(b)** Corrupted signal.

Stress wave signals were induced by striking a *Cinnamomum camphora* sample which is shown in Figure 3. The diameter was 27 cm. The signals were measured using a piezoelectric transducer (Beidaihe Institute of Electrical Automation, Model: BZ1106A) and a DAQ instrument (National Instruments, Model: USB-6259) with a sampling frequency of 100 KHz. A typical signal recorded and its spectrum were shown in Figure 4(a). Additive Gaussian white noise, generated by MATLAB code awgn.m, was used as the noise source embedded in the stress wave signal. The contaminated signal with SNR value of 0 dB was depicted in Figure 4(b).

### 4.2. Performance Evaluation with Stress Wave Signals

**Figure 5.**
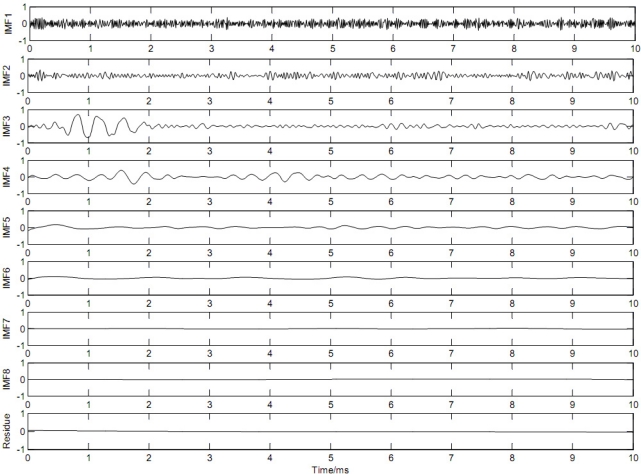
The stress wave signal as shown in Figure 3(b) is decomposed into eight IMFs (IMF1-IMF8) and one residue using EEMD.

First the EEMD algorithm was applied to the contaminated signal as shown in Figure 4(b). The parameters used to run the EEMD algorithm were trial number and added noise power, which were set 100 and 0.2 times the standard deviation of the contaminated signal, respectively [18]. Figure 5 displays a sequential extraction of oscillations by EEMD. The EEMD decomposed the noisy signal into 8 IMFs and a residual. One can remark that the first IMF corresponds to a fast oscillation, whereas the 8th corresponds to a slow one.

**Figure 6.**
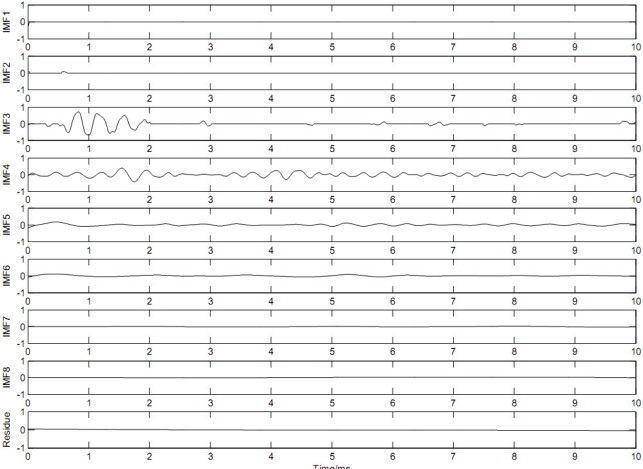
Results obtained by the threshold operation according to IHP.

Then the zero-crossings of each IMF were located and the IHPs were calculated according to Equation (3). The noise dominated oscillations were removed by the threshold operation using Equation (4), where the parameter *α* is set to 1.0. Figure 6 shows the restored IMFs. Like the EMD-based low pass filter [12], the low order IMFs are expected to be noise-only IMFs and the high order IMFs signal-only IMFs. As shown in Figure 6, IMF1 was set to zero and the higher order IMFs, IMF4-IMF8, were retained without any change. However, IMF2 and IMF3 contain both signal structures and noise structures which can neither be set to zeros nor be retained without any processing. Thus, in this study, we retrieve the most important structures of signal from its noisy version according to the IHP value. The oscillations with large IHP were retained and those with shorter IHP were set to zeros.

**Figure 7.**
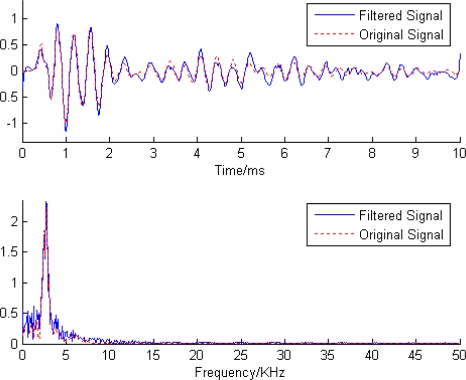
The denoised stress wave signal using proposed method.

Finally, the filtered signal was reconstructed according to Equation (5). Figure 7 shows the outcome of applying the proposed filtering scheme to the noisy signal.

As expected, the noise had been effectively reduced. A significant result (solid line) was obtained which was very close to the original signal (dot line). The reconstruction of proposed method jumped where the original signal jumped and was smooth where the original signal was smooth.

**Figure 8.**
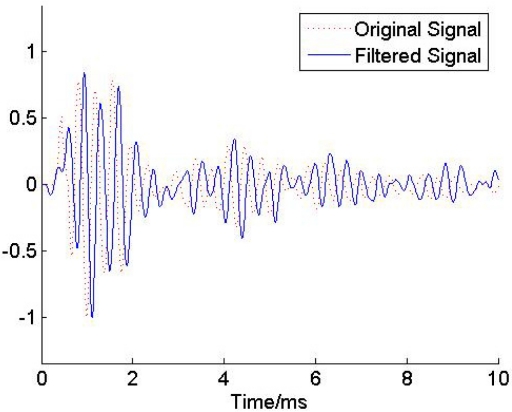
The denoised result using Butterworth low pass filter.

A Butterworth low pass filter with an experimentally identified 6th order was used to filter the stress wave signal. The cut off frequency was set to 5 KHz. The result is shown in Figure 8. We see that the low pass filter can be used to reduce the noise contamination of the data. However, comparing the original signal and filtered signal as shown in Figure 8, we find that a big phase delay was induced. This is mainly due to the fact that the phase shift is unavoidably used during the filtering process. In general, a high order results in a big phase delay.

We also added two results of well-known denoising methods: EEMD-based low pass filter and EEMD-based thresholding filter. For the low pass method, the noisy signal was decomposed into several IMFs at first. IMF1 and IMF2 were considered as noise-only IMFs which were removed and not used in the reconstruction. In the case of EEMD-based thresholding filter, all IMFs were shrinkaged by a soft function given in the following Equation [14]:

(8) $${\hat{c}}_{i}(k)=\{\begin{array}{cc} c_{i}(k)-\lambda , & c_{i}(k)\geq \lambda \\ 0, & |c_{i}(k)|<\lambda \\ c_{i}(k)+\lambda , & c_{i}(k)\leq -\lambda \end{array}$$

Where *λ* denoted the threshold value proposed by Donoho and Johnstone [9].

**Figure 9.**
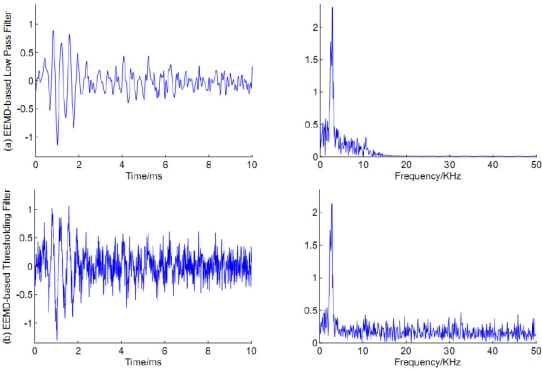
Filtered results using EEMD-based low pass filter (top) and EEMD-based thresholding filter (bottom).

The denoising results using EEMD-based low pass filter and EEMD-based thresholding filter are shown in Figure 9. It is obvious that EEMD-based thresholding filter got a worse filtering result. As can be observed from the spectrum of the filtered signal, the EEMD-based low pass filter can eliminate the noise efficiently when the frequency is above 10 KHz. However, it can’t work well when the noise overlaps a bandwidth from 5 KHz to 10 KHz. Comparing Figures 7 and 9, it is clear that the proposed approach was applied successfully to reduce noise and achieved better filtering performance.

**Figure 10.**
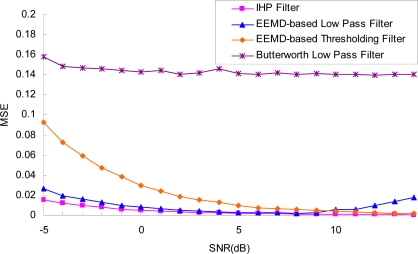
MSE obtained with different noise levels by proposed method, EEMD-based low pass filter and EEMD-based thresholding filter.

We demonstrated the effectiveness of presented method with different noise levels. The SNR ranged from −5 to 15 dB. Figure 10 shows the performance under different noise levels with the two EEMD-based methods mentioned above.

The performance of denoising can be evaluated by the objective measures of mean square error (MSE) between filter output and original signal, which is defined as in Equation (9):

(9) $$\mathrm{MSE}=\frac{\sum_{k=0}^{L-1}{\left[x(k)-\hat{x}(k)\right]}^{2}}{L}$$

In the Equation, *x*(*k*) and *x̂*(*k*) denote the values of the original signal and restored signal, respectively, and *L* is the original signal’s length. Generally, under given noise variances, the lower MSE value represents better filtering performance. In this paper, the MSE is an average of 10 times repetitions.

It is evident that our method is capable of producing better noise-removal results throughout the whole input SNR range. We would like to stress here that the noise can be reduced effectively (MSE obtained is 0.02) even in cases where the signal quality is low (SNR value is −5 dB). This means that the method is effective for very noisy signals. For the EEMD-based low pass filter, the performance is affected by the input global SNR. When the SNR is small, IMF1 and IMF2 are noise-only IMFs, and the noises can be eliminated effectively by removing the IMF1 and IMF2. With the decrease of the noise level, the signal is rather “clean”. IMF1 and IMF2 are dominated by signal structures. Removing the two IMFs will result in removal of the signal-dominated oscillations. The EEMD-based thresholding filter cannot reduce the noise when the SNR is small, where the signal quality is low, so the thresholding filter is not suitable for detecting the target signals submerged in strong noise. For the Butterworth low pass filter, the value of MSE is big and does not change from −5 dB. Here, the big MSE is probably not due to poor noise reduction performance, but rather to the phase delay.

## 5. Conclusions

In this paper, a novel denoising method using EEMD and IHP model for stress wave signals corrupted with additive Gaussian noise is proposed. The principle in this approach is that the noises usually occupy the high frequency band, *i.e.*, the periods of the noise structures are usually shorter than the periods of signal structures. Thus, one can detect and remove the noise-oscillation within each IMF rather than remove the noise-only IMF as in the EEMD-based low pass method. The results in this work show that the proposed method can be applied to enhance stress wave signals, even in cases where the signal quality is low (SNR value is −5 dB). This method has given a better performance compared to the Butterworth low pass filter, EEMD-based low pass filter and EEMD-based thresholding filter.

The main disadvantage of this method is that, because the current status of the EEMD still lacks and theoretical grounds, the present study has been conducted on the basis of extended numerical experiments. In future work, we plan to test the method on more signals acquired directly from different devices and in different experimental conditions such as noise levels, sampling rates, and sample sizes.
